# Letter on: “Electrophysiological and Biochemical Evaluations of Neuropathy Risk in Oral Levodopa versus Levodopa/Carbidopa Intestinal Gel Treatment”

**DOI:** 10.1055/s-0046-1825516

**Published:** 2026-07-29

**Authors:** Osheen Bhandari, Prishita Kumar, Sajjan Pal, Sandeep Singh Saini

**Affiliations:** 1 Christian Medical College and Hospital, College of Physiotherapy, Department of Physiotherapy, Ludhiana PB, India.; 2 Maharishi Markandeshwar (Deemed to be University), Maharishi Markandeshwar Institute of Physiotherapy & Rehabilitation, Department of Physiotherapy, Mullana HR, India.

Dear Editor,

We read with great interest the article titled “Electrophysiological and biochemical evaluations of neuropathy risk in oral levodopa versus levodopa/carbidopa intestinal gel treatment.” 1 The authors compared biochemical and electrophysiological parameters in patients receiving oral levodopa and levodopa/carbidopa intestinal gel (LCIG). The topic is clinically-important because peripheral neuropathy is a relevant non-motor complication in advanced Parkinson's disease and may influence therapeutic decisions. The inclusion of metabolic markers such as vitamin B12, folate, and homocysteine alongside nerve-conduction studies provides a comprehensive attempt to explore mechanisms underlying neural dysfunction.

The authors appropriately discuss gastrointestinal dysfunction in advanced disease and the pharmacokinetic benefits of continuous intestinal infusion. Studying subclinical electrophysiological abnormalities together with biochemical parameters may help early detection of neurological complications. However, several methodological and interpretative aspects warrant further clarification.

First, although the study aimed to evaluate neuropathy risk, it employed a prospective cross-sectional design. Such a design identifies associations but cannot establish temporal relationship or causality between treatment modality and neuropathy development. Therefore, the inference of ‘risk’ may be stronger than the evidence supports, and longitudinal follow-up would be required to determine whether LCIG independently contributes to neuropathy progression. 2

Second, the small sample size ( *n* = 32) and unequal group distribution limit statistical power and generalizability. Importantly, the LCIG group required a significantly-higher daily levodopa dose and levodopa equivalent daily dose (LEDD). Because levodopa metabolism increases homocysteine in a dose-dependent manner, biochemical and electrophysiological differences may reflect cumulative pharmacological exposure rather than route of administration. Without regression modelling or dose-adjusted analysis, it is difficult to attribute findings specifically to LCIG. 3

Third, nerve-conduction studies were performed unilaterally, and abnormalities were determined using laboratory reference values. Polyneuropathy is typically a symmetrical length-dependent process, and unilateral testing may underestimate or misclassify neuropathy. Moreover, no standardized diagnostic criteria or validated clinical neuropathy scale was applied. Therefore, the findings represent parameter-level variations rather than confirmed neuropathy. The reporting of absent sural responses without classification into diagnostic categories further limits clinical interpretation. Consequently, the results appear to indicate subclinical electrophysiological variability rather than definite polyneuropathy. 4

Additionally, clearer adherence to established observational reporting standards such as those of the Strengthening the Reporting of Observational Studies in Epidemiology (STROBE) statement would improve transparency. Important details including predefined outcomes, confounder adjustment strategy, and handling of multiple comparisons were not fully described. Explicit reporting of neuropathy diagnostic criteria would enhance reproducibility and interpretability. 5

Finally, the conclusions appear stronger than the presented data support. The study demonstrates biochemical alterations and electrophysiological differences, but it infers neuropathogenic effects of LCIG therapy. Considering the cross-sectional design, absence of standardized neuropathy diagnosis, and markedly-higher levodopa exposure in the LCIG group, the findings are more appropriately interpreted as associations involving cumulative levodopa burden, metabolic imbalance, and subclinical nerve-conduction changes rather than evidence of a formulation-specific complication.

In summary, this study provides useful preliminary observations regarding metabolic and electrophysiological changes in advanced Parkinson's disease therapy. However, longitudinal studies incorporating standardized neuropathy criteria, dose-adjusted analyses, and structured reporting are required before concluding that LCIG independently increases neuropathy risk. Clarification of these aspects would improve clinical applicability and guide monitoring strategies for patients receiving long-term levodopa treatment.

## References

[1] Erdem M Selluncak B Balal M Fidanci H Demirkiran M Electrophysiological and biochemical evaluations of neuropathy risk in oral levodopa versus levodopa/carbidopa intestinal gel treatment Arq Neuropsiquiatr 2025 83 12 1 7 10.1055/s-0045-1813243 PMC12685543 41360401

[2] Mann C J Observational research methods. Research design II: cohort, cross sectional, and case-control studies Emerg Med J 2003 20 01 54 60 10.1136/emj.20.1.54 12533370 PMC1726024

[3] Müller T Levodopa-related peripheral neuropathy: clinical aspects and management Drugs Aging 2013 30 11 915 921 10.1016/j.parkreldis.2013.02.006

[4] American Academy of Neurology England J D Gronseth G S Franklin G Practice Parameter: evaluation of distal symmetric polyneuropathy: role of autonomic testing, nerve biopsy, and skin biopsy (an evidence-based review) [RETIRED]. Report of the American Academy of Neurology, American Association of Neuromuscular and Electrodiagnostic Medicine, and American Academy of Physical Medicine and Rehabilitation Neurology 2009 72 02 177 184 10.1212/01.wnl.0000336345.70511.0f 19056667

[5] STROBE Initiative Von Elm E Altman D G Egger M Pocock S J Gøtzsche P C Vandenbroucke J P The Strengthening the Reporting of Observational Studies in Epidemiology (STROBE) statement: guidelines for reporting observational studies PLoS Med 2007 4 10 e296 10.1371/journal.pmed.0040296 17941714 PMC2020495

